# A sampling strategy for genome sequencing the British terrestrial arthropod fauna

**DOI:** 10.12688/wellcomeopenres.18925.1

**Published:** 2023-03-20

**Authors:** Liam Crowley, Heather Allen, Ian Barnes, Douglas Boyes, Gavin R. Broad, Christopher Fletcher, Peter W.H. Holland, Inez Januszczak, Mara Lawniczak, Owen T. Lewis, Craig R. Macadam, Peter O. Mulhair, Lyndall Pereira da Conceicoa, Benjamin W. Price, Chris Raper, Olga Sivell, Laura Sivess

**Affiliations:** 1 Biology, University of Oxford, Oxford, Oxfordshire, OX1 3SZ, UK; 2 Natural History Museum, London, SW7 5BD, UK; 3 UK Centre for Ecology & Hydrology, Wallingford, OX10 8BB, UK; 4 Tree of Life, Wellcome Trust Sanger Institute, Hinxton, CB10 1SA, UK; 5 Buglife - The Invertebrate Conservation Trust, Peterborough, PE2 8AN, UK

**Keywords:** Earth BioGenome Project, Invertebrate, Whole-Genome Sequencing, Collecting, Preservation, Prioritisation

## Abstract

The Darwin Tree of Life (DToL) project aims to sequence and assemble high-quality genomes from all eukaryote species in Britain and Ireland, with the first phase of the project concentrating on family-level coverage plus species of particular ecological, biomedical or evolutionary interest. We summarise the processes involved in (1) assessing the UK arthropod fauna and the status of individual species on UK lists; (2) prioritising and collecting species for initial genome sequencing; (3) handling methods to ensure that high-quality genomic DNA is preserved; and (4) compiling standard operating procedures for processing specimens for genome sequencing, identification verification and voucher specimen curation. We briefly explore some lessons learned from the pilot phase of DToL and the impact of the Covid-19 pandemic.

## Introduction

The Darwin Tree of Life (DToL) programme aims to sequence and assemble genomes of all British and Irish eukaryote species. Around 70,000 species have been recorded in Britain and Ireland, although some of these will be accidental introductions, rare vagrants or no longer resident. Work is needed to refine the current species list. Even so, a large percentage (∼40%) of this fauna and flora comprises terrestrial and freshwater arthropods. Given the significant proportion of species accounted for by this group, an effective sampling strategy is fundamental within any project seeking to sequence a complete eukaryotic biota.

Darwin Tree of Life is part of the Earth BioGenome Project (EBP) ( Lewin *et al.*, 2018). Since 2020 a consortium of UK partners have been working on the pilot project, with the rationale and aims outlined by The Darwin Tree of Life Project Consortium (2022). The initial goal of the pilot phase was to produce 2,000 assembled genomes, with each genome being derived from one specimen whenever possible, although this numerical goal was impacted by restrictions imposed by the Covid-19 pandemic. This phase of DToL is producing a large genomic resource but also developing methods and embedding best practices that benefit other EBP projects.

Due to the variation and spread of expertise across taxa, the DToL project is organised into a series of taxon-specific working groups. Here, we focus on sampling strategy ( Figure 1) and consider the lessons learned with regards to sampling terrestrial and freshwater arthropods. Much will be relevant for any efforts towards large-scale sequencing projects. Throughout, the term ‘arthropod’ is used as short hand for ‘terrestrial and freshwater arthropods’.

## Methods

**Figure 1.**
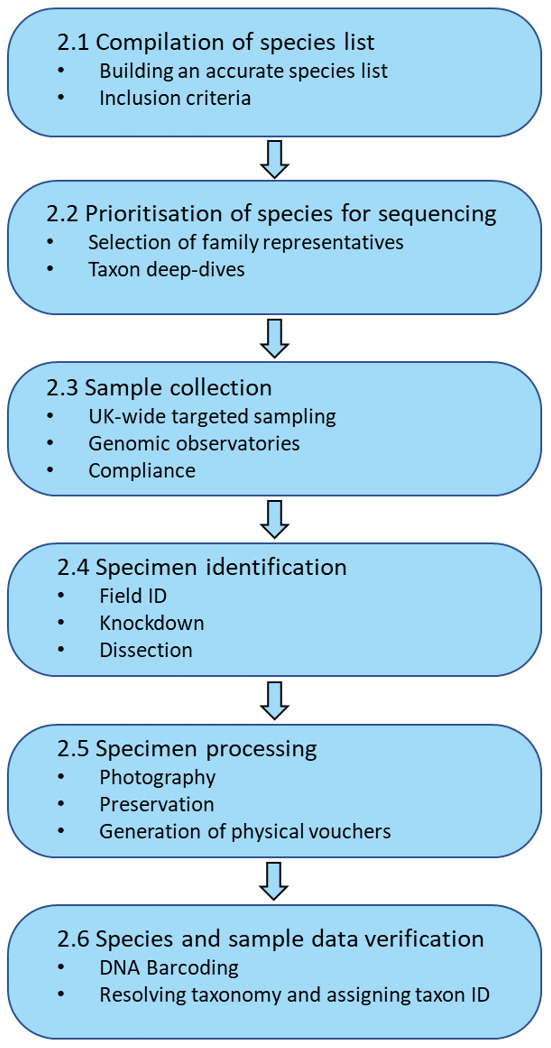
Flow diagram outlining the complete sampling process -

### Compilation of the species list

***Deriving accurate species counts for Britain and Northern Ireland.*** National species lists are essential to target collecting and to measure progress. A species list also needs to be partitioned into a taxonomic hierarchy; sampling in DToL is in part hierarchically structured, aiming to collect as many family representatives as possible in the first phase, genera in the second phase, and with more complete species representation for some key groups.

***Criteria for inclusion in DToL.*** A species is within scope of DToL if it is established outdoors in a wild state. As well as native species, this means that various established non-natives will be sequenced, but not species established only indoors, in glasshouses, warehfouses, and similar. The main source of native status is the GB Non-native Species Information Portal. Occasional immigrants or occasional importations are also not considered, although these have been collected *ad hoc*, for potential future sequencing. The source of a particular specimen, however, could be a laboratory colony if the species satisfies the criteria. This produces a working list of species that have extant populations in Britain and Ireland. Whilst this restriction whittles down the bird list substantially, it has limited impact on the arthropod list.

The UK Species Inventory ( UKSI) is maintained at the Natural History Museum and is the most comprehensive, curated source of species names for biological recording in the UK. The UKSI database forms the foundation for the largest biological recording and reporting systems in the UK, including the National Biodiversity Network (and all partners included within) ( Raper, 2021). The Inventory functions to hold a standard checklist of taxa so that records can be made against them and allows for cross-referencing between records to account for the same species that is known by different names. It acts as an archive of checklist editions, allowing users to search names (and other information on taxa such as conservation status) and how they have changed through time. These features are critical for sound reporting, ensuring that all available data are available for a taxon despite the variation in names used over time. 

The UKSI works closely and continuously with a network of dedicated professional and amateur biological recorders (as coordinated by the Biological Records Centre) and taxonomists, other databases ( *e.g.*, GBIF, WoRMS) and published checklists to ensure taxonomic concepts are up to date. Species across all forms of cellular life that have been recorded in the UK and Ireland, or for which there is a potential need to record, can be included in UKSI. Because the UKSI serves primarily as a resource for recording, it cannot be used as the default checklist for DToL without modification, as species native or established in Britain and Ireland form only (a large) part of the checklist.

An important task is therefore to create a curated, baseline checklist of UK species to track sampling progress against. Developments to create a UKSI API are underway by the NHM, from which to draw live UKSI checklists with updating taxonomy which will be relevant for future biodiversity projects.

**Figure 2.**
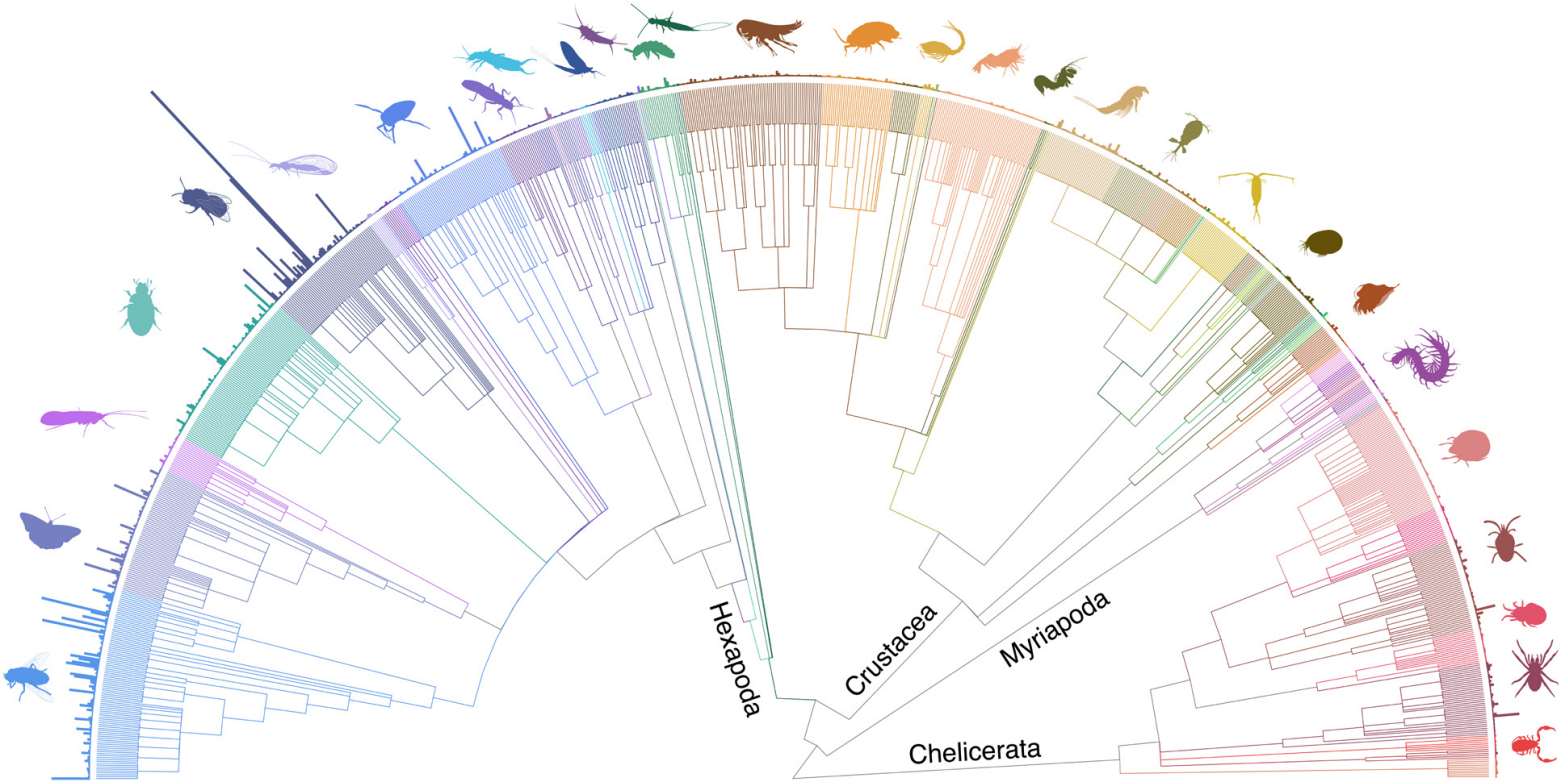
Tree of UK arthropod families with annotation of species count per family.

The total number of species (all life) listed in the UKSI, and therefore available to record, currently stands at 76,059. Excluding non-natives this total is reduced to 72,512 ( Figure 2).

The following two examples are used to demonstrate how species lists are refined from the raw lists within UKSI to the target lists for DToL t: (1) the list of British and Irish butterflies, and (2) the families of Hymenoptera.

(1) UKSI includes the names of 192 species of butterflies (Lepidoptera, families Hesperiidae, Lycaenidae, Nymphalidae, Papilionidae, Pieridae and Riodinidae). Of these, 122 species have been included in standard British and Irish Lepidoptera checklists ( *e.g.*, Agassiz *et al.*, 2013). The remaining 70 names are in UKSI but have not been considered part of the UK fauna; they are present in UKSI for a variety of reasons, including misidentifications, listings in legislation, among others. A total of 49 species are included in Agassiz *et al.* (2013), but on the basis of presumed introductions or importations, or with a lack of evidence as to how they came to be collected (or reported as collected) in Britain. Three species are extinct in Britain and Ireland with one additional species ( *Heteropterus morpheus*) listed as an extinct resident of the Channel Islands, and therefore not within the scope of DToL. A further 10 species are scarce or very scarce migrants and thus not within scope of DToL. One species ( *Nymphalis polychloros*) might be in the early stages of recolonising, but is not firmly established. This leaves 59 butterfly species considered to be within the scope of DToL. One of these has been reintroduced and some others are rare and protected, so specimens of some species have been sourced from outside of Britain and Ireland.
(2) There are 73 families of Hymenoptera on the British and Irish checklist, taking into account some recent splits within the superfamily Apoidea ( Sann *et al.*, 2018). Of these, the species of three families (Eucharitidae, Megalodontesidae, Orussidae) are considered extinct in Britain and Ireland, if indeed they ever naturally occurred here ( Dale-Skey *et al.*, 2016; Liston *et al.*, 2014). None of the species in 18 families reach the size threshold (around 5 mm) for the pilot phase of DToL so this leaves 52 families within scope of this phase of DToL and therefore to be prioritised for collecting and sequencing at least one species of each family.

### Prioritising species for sequencing

As it is not possible to collect and sequence every species immediately, it is necessary to identify certain species as targets and prioritise their collection and processing into the sequencing pipeline. The DToL project is divided into three phases, with the first phase focusing on achieving taxonomic breadth via aiming to sequence one or two species from every family (if individuals are large enough) present in Britain and Ireland. Approximately 40% of global terrestrial and freshwater arthropod families are represented by species in Britain and Ireland ( Table 1), so a lot of higher-level arthropod diversification is potentially covered by DToL, with some exceptions, such as scorpions, phasmids and millipedes. The species identified from each family are considered ‘family representatives’, and are selected following a criteria hierarchy described below. It is acknowledged that for a number of families in the checklist it will be very difficult to obtain specimens for sequencing. This will be offset by sequencing additional species for other families. Some families have been selected for taxonomic ‘deep dives’, with multiple species selected and prioritised following an equivalent criteria hierarchy.

**Table 1.** Numbers of families per arthropod order with terrestrial or freshwater species, globally and in Britain and Ireland.

Insecta			Arachnida			Crustacea			Entognatha			Myriapoda		
order	GBI families	global families	order	GBI families	global families	order	GBI families	global families	order	GBI families	global families	class	GBI families	global families
Coleoptera	103	190	Sarcoptiformes	106	266	Isopoda	13	61	Poduromoprha	6	11	Diplopoda	17	148
Diptera	106	167	Trombidiformes	73	151	Amphipoda	5	49	Symphypleona	7	10	Chilopoda	11	16
Lepidoptera	71	137	Mesostigmata	41	130	Cladocera	10	24	Diplura	1	10	Pauropoda	2	12
Hemiptera	60	126	Araneae	33	129	Decapoda	6	14	Entomobryomorpha	5	8	Symphyla	2	2
Hymenoptera	73	108	Opiliones	6	45	Podocopida	7	13	Protura	3	7	**Total**	**32**	**178**
Psocodea	30	103	Pseudoscorpiones	6	24	Harpacticoida	8	10	Neelipleona	0	1			
Trichoptera	19	47	Scorpiones	1	22	Anostraca	1	8	**Total**	**22**	**47**			
Orthoptera	8	37	Solifugae	0	12	Calanoida	4	6						
Odonata	9	27	Amblypygi	0	5	Cyclopoida	4	4						
Ephemeroptera	10	23	Metastigmata	2	3	Anaspidacea	0	4						
Siphonaptera	7	18	Palpigradi	0	2	Siphonostomatoida	3	3						
Neuroptera	6	16	Schizomida	0	2	Bathynellacea	1	2						
Plecoptera	7	15	Ricinulei	0	1	Cephalobaenida	2	2						
Blattodea	5	15	Thelyphonida	0	1	Notostraca	1	1						
Mantodea	0	15	**TOTAL**	**268**	**793**	Mysida	1	1						
Dermaptera	4	14				Arguloidea	1	1						
Phasmatodea	3	14				Gelyelloida	0	1						
Embioptera	0	11				Poecilostomatoida	1	1						
Thysanoptera	4	10				**TOTAL**	**68**	**205**						
Strepsiptera	1	9												
Mecoptera	2	9												
Zygentoma	1	5												
Archaeognatha	1	2												
Raphidioptera	1	2												
Archaeognatha	1	2												
Megaloptera	1	2												
Zoraptera	0	2												
Notoptera	0	2												
												**TOTAL** **GBI** **families**		**922**
**TOTAL**	**532**	**1126**										**TOTAL** **global** **families**		**2349**

***Selection of family representatives.*** A species selection hierarchy was employed to identify the most suitable candidates to act as family representatives for each taxonomic family. Of 1,352 arthropod families, 494 contained just one or two species, therefore these species were automatically selected as the family representatives.

The first and foremost criterion in the hierarchy for selecting representative species was the indication that there was a need and/or desire for this genome from the scientific community. This need/desire was established via a community consultation using a short survey distributed through research networks. This survey is still open, but now the project primarily seeks suggestions for any British and Irish eukaryote species through a separate survey. During the first year of this consultation, species from a total of 90 different arthropod families were suggested, of which 83 were selected as family representatives. Some examples of species suggested in the survey that were collected and are now proceeding through the sequencing pipeline include *Ammophila sabulosa, Bombus lapidarius, Ixodes ricinus, Operophtera brumata,* various Syrphidae *, Braula coeca, Agelastica alni, Halyzia sedecimpunctata* and *Lampyris noctiluca*.

The remaining (905) families, that were neither monospecific/dispecific nor suggested in the community consultation, were assessed against the following criteria to identify appropriate representative species:

- Important/Iconic/Interesting species. Species known to be of particular interest due to economic, social, ecological or evolutionary factors. For example, those used as model species in laboratory or field research. Such species may be indicated by a relatively extensive presence in the scientific literature.
- Availability/ease of collecting a specimen. Species that are more easily available to collect than others in the family. This may include species that are more common, widespread, abundant, phenologically wide-ranging or present at a genomic observatory site.
- Ability/ease of accurate identification. Species that may be more readily identified than others in the family, especially those which can be reliably determined in the field.
- Larger body size. Species with a greater body mass, thereby a single individual providing more tissue and therefore DNA for extraction.
- Pre-existing genetic data. Species for which there is existing, publicly available genetic data that would increase the value and utility of sequence data. For example, species that have a publicly available transcriptome.

***The value of ‘taxon deep dives’.*** Some taxa are of particular ecological/evolutionary interest, whereby it is desirable to sequence at a greater resolution than one or two species per family early on in the project. These taxa were selected as ‘deep dives’ whereby many or all species within the group were targets for prioritised sequencing. The deep dive groups were selected using a similar criteria hierarchy to the family representatives:

- Important, iconic or interesting group. The taxon as a whole is of particular interest due to economic, social, ecological or evolutionary factors. For example, the taxon may contain several species of interest or species across a diverse range of traits for one of these factors.
- Species pairs for phylogenetic analysis. Taxa that contain pairs or paired groups of species that possess both alternative phenotypes of a trait of interest.
- Taxon sets from Genomic Observatory. The full set of a taxon from a genomic observatory site.

The taxonomic level of the deep dive may vary depending on the number of extant species represented domestically, and the reason the group was selected. Broad groups will include too many species to remain practical and narrow groups will not include enough species to be informative. Such deep dives provide advantages that sparser sampling does not. For example, ensuring phylogenetic coverage of the group, including basal species. Examples of taxa identified for deep dives include: Lepidoptera, Syrphidae, bees (Apoidea: ‘anthophila’) and Coccinellidae.

***Sample collection.*** Sample collection is a continuous process following one of two approaches: targeted collections guided by the species priority list, or site-specific general sampling of all species. Targeted collections are facilitated by the maintenance of lists which are as current as possible, so that all partners and collaborators can check whether any particular species has been collected, and how many specimens have been collected. Amalgamating lists through a common portal is a major challenge which we are working on.

***UK-wide targeted sampling.*** Targeted sampling of species identified as family representatives were made either during specific collecting trips or by expert volunteers and sent via a postal service to a genome acquisition laboratory. We have been able to tap into existing activity, such as annual field meetings of the Dipterists’ Forum or the British Myriapod and Isopod Group (BMIG), where experts in the identification of various groups of invertebrates are brought together on one site and have given very generously of their time and expertise. Non-Taxon specific “Bioblitzes”, usually organised as a tool for biological monitoring, bring taxonomic experts together in a similar fashion. Similarly, identification experts around the country, often ‘amateur’, have enthusiastically sent specimens of interest, either under the auspices of a recording scheme or society ( *e.g.*, the British Arachnological Society), or as individuals.

This sampling approach allows relatively efficient gathering of specimens of species that are geographically restricted or specialised within habitats not covered by site-based collecting. It also has advantages in engaging taxonomic, often amateur, experts across a wide range of taxa which has the dual benefit of providing specimens that would otherwise not have been possible to obtain whilst simultaneously engaging this community, including recording schemes and societies, in the importance and value of genomic collections. 

The spatially and temporally diffuse and sporadic nature of this approach, however, also carries a number of disadvantages that require carefµL consideration to overcome. The first disadvantage relates to a potential for loss of specimen quality, for example from breaks in the cold chain ( *e.g.* dry shipper failing) or premature mortality of specimens ( *e.g.* specimens dying during shipping before arriving at laboratory facility for flash-freezing - although worth noting any specimens which are ‘dead on arrival’ can be preserved in ethanol and used for future DNA barcoding). Widespread sampling also inherently carries a greater degree of logistical challenges, including the need for a greater amount of documentation ( *e.g.* many individual collection permits and Material Transfer Agreements for accessioning in the NHM’s collection) and greater time and financial cost per specimen.

***The value of intensive site-based collecting (Genomic observatories).*** The other approach taken was site-base collections. Specific sites were selected based on factors such as the diversity and representativeness of species present, geographic location and the availability of laboratory facilities. The first site selected was Wytham Woods and the wider Wytham estate, owned by the University of Oxford. Wytham has been designated as a ‘genomic observatory’, with the aim of sequencing every species present at the site.

This approach has several distinct logistical advantages including:

- simplified cold chain and access to labs on site;
- year-long collecting overcoming phenological limitations;
- Simpler and streamlined arrangements for permissions and permits;
- The potential for local rearing facilities ( *e.g.* for parasitoids, with host material from the focal site supplemented by material sourced more widely);
- if the site had a history of biological recording or ecological study, this pre-existing knowledge was valuable in developing a target list for collections;
- economies of scale and practicalities of the processing chain: field collections are processed and shipped in fewer batches;
- intensive sampling is often the only way of collecting infrequently encountered species ( *e.g.* species that exist at low population densities or that have adult/accessible life stages for a short time period;
- repeat collections can be easier (sequencing failures or deficiencies mean that some specimens need to be re-collected).

Site-based collections are also less targeted towards priority species, instead aiming to simply collect everything that can be accurately and appropriately identified and processed. This allows for more efficient collecting at scale, with a diminished need to prepare and collect lists.

The site-based collecting approach, however, also has certain disadvantages. One such significant disadvantage is that despite the broad taxonomic spread at any site (only diverse and representative sites were selected), the composition of species present at any given site will only be a limited sample of national biodiversity dependent on the habitats present, region, site history, among others. This disadvantage can be partly offset by the selection of multiple sites with complementary faunas and floras so as to minimise sampling redundancy. Being spatially restricted also limits the pool of expertise that can be accessed, as it is likely that only local experts will be available to assist with collections. This may also introduce a bias into the taxa collected reflecting the local expertise rather than the true distribution of species.

**Figure 3.**
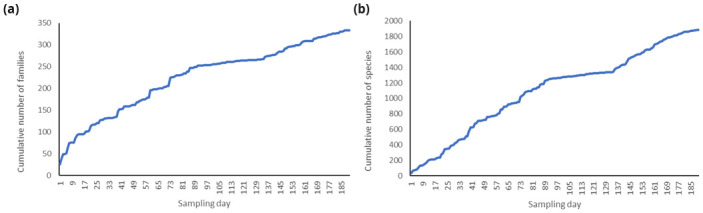
Accumulation curves of the cumulative number of families ( **a**) and cumulative number of species ( **b**) collected against the first 190 sampling days at the Wytham Woods genomic observatory.

***Overview of sampling progress.*** Over the course of phase 1, collections were targeted to the priority species as identified in the prioritisation process (2.2). Due to the projected scaling up of sequencing throughput by the project, it was necessary for collections to outpace the initial sequencing rate. This necessitated collections beyond simply just priority taxa, with as many available taxa collected as possible alongside targeted collections to ensure that priorities were covered. In total, 7891 arthropod specimens were collected over the course of phase 1 (2019-2022), representing 4969 species of 1087 families ( Table 2). By examining species and family accumulation curves for a specific site, Wytham, the rate of new taxa collection can be assessed ( Figure 3). Such accumulation curves show that genomic observatory based collections continue to produce novel species. Changes in the rate of novel taxon acquisition also indicate the development of sampling practice. For example, after sampling day 130 at Wytham Woods, an integration of expert amateur natural history group collection events and local volunteer collectors led to an increase in the rate of novel taxon acquisition.

**Table 2.** Total number of arthropod specimens/species/families collected each year by UK-wide sampling (as adopted by the Natural History Museum) versus Wytham Woods site-based sampling. Totals per year are the number of new taxa for the project collected by the sampling approach.

Year	UK-wide targeted sampling	Site-based sampling
2019	88/66/30	317/221/99
2020	514/316/123	691/546/72
2021	1680/1069/258	960/547/92
2022	2621/1634/343	1020/570/70

***Legal and Ethical issues, A.K.A. ‘compliance’.*** Within the UK, very few arthropod species are protected by law. Species protected under Schedule 5, section 9.4a of the Wildlife & Countryside Act 1981 include two spiders, three freshwater crustaceans and 25 insects; a small subset of these are also protected under international conventions. For these species, special permission from DEFRA must be sought to collect specimens. Collecting on National Nature Reserves and Sites of Special Scientific Interest (SSSIs) requires permission from the relevant statutory agency (Natural England, Nature Scot, Natural Resources Wales, or the Council for Nature Conservation and the Countryside (Northern Ireland)), and these agencies have been very supportive of DToL. Collecting on privately-owned land requires the landowner’s permission, in addition to permissions from government agencies if this land has statutory protection.

Specimens being acquired by the Natural History Museum and other museums require evidence that they were collected legally and ethically. Typically, an acquisition is accompanied by a Material Transfer Agreement or, for staff collections, a staff collection form and/or collection enhancement form. Additionally, prior to specimens being sent to Sanger, the specimen manifest must pass legal compliance tests.

DToL partners have signed up to a code of conduct within the project and we ensure that arthropod sampling follows the Code of Conduct for Collecting Insects and Other Invertebrates ( Invertebrate Link, 2002).

***Specimen identification.*** Most arthropod specimens are entirely destroyed for DNA extraction so confidence in the identification is essential; when the identification is queried there will usually be no recourse to a specimen, although there are exceptions (see below). Several factors increase our confidence in the identification being accurate: (1) trust in the collector’s/submitter’s identification skills; (2) voucher photographs; (3) independent DNA barcoding, matching the specimen’s barcode sequence to a reliable barcode sequence in BOLD or NCBI; (4) in some cases, features associated with identification *e.g.* genitalia and/or wings can be retained as morphological vouchers; (5) a stable taxonomy for the genus.

For various groups, particularly more mobile insects, it is often necessary to anaesthetise the specimen (using either CO2 or ethyl acetate) to check critical features for identification. In addition to photographs of the specimen alive, it is again photographed when about to be processed on dry ice. Photography with a macro lens at this stage can provide very usefµL diagnostic photos. In some cases it is difficult or impossible to take images of live specimens or specimens dead on dry ice in the orientation necessary to see critical features; it is also impossible for anybody processing samples to know exactly which features are critical for every species, although the taxon-specific SOPs provide as much guidance as is feasible at the taxonomic level of order. It can also be very difficult to see certain anatomical features on a live insect even when these can be obvious on a pinned specimen in a collection. For various groups, it is possible to retain parts of specimens to serve as morphology vouchers. We have been retaining wings of macrolepidoptera and genitalia of several insect groups, particularly various moths, some Diptera (such as Mycetophilidae) and certain genera of Hymenoptera ( *e.g.*, *Netelia*). These parts are removed when the specimen is placed on dry ice for processing.

DNA barcoding is an important extra verification step and has been invaluable in pointing to misidentifications in some difficult groups. It is important to note though that (a) verification is only as good as the reference barcode sequences, and (b) DNA barcoding using standard markers does not reliably differentiate species of all genera ( *e.g.*, certain ichneumonid wasp genera: Klopfstein *et al.*, 2016). Barcode verification is explored in more detail below. Specimens collected for DToL have not all been stored for genome sequencing; some have been retained as vouchers for DNA barcoding or morphology. Development of reference collections and reference barcode libraries goes hand in hand with DToL and other genomics projects.

Many identifications of specimens destined for genome sequencing are provided by experts from outside of the formal DToL partners. These experts can be professional, including consultants, or amateur. Much of the UK’s taxon identification expertise lies within the ‘amateur’ community and a project such as DToL relies on this dedicated army.

### Specimen processing, preservation and archiving

For optimal DNA preservation the invertebrate specimen has to be flash-frozen live at temperatures of -80°C or lower. This can be achieved either by keeping specimens alive until freezer facilities are available or, in the field, using portable freezing methods such a liquid Nitrogen dry shipper, or dry ice. From the point of freezing, the ‘cold chain’ of the specimen must be maintained. This means that the specimen must remain at -80°C or below throughout sample processing and sample shipment.

***Unique identifiers and sample data.*** Before processing, each specimen is assigned a unique identifier, *e.g.* a unique specimen ID (UID). Samples being sent for whole genome sequencing (in this case, to the Sanger Institute) are accompanied by a sample manifest; data fields and data standards for the manifest are covered by Lawniczak *et al.* (2022) but, in brief, this includes metadata pertaining to species concept identity, collector information, collection details, tissue sample details, among others. Data management for large collections and herbaria (the Natural History Museum in this context) can be complex, with specimens and tubes needing to be tracked across the Collection Management System, freezer storage software and the sample manifest as errors are difficult to correct further down the line, once specimen and DNA data have been promulgated across systems.

***Sample processing - overview.*** All samples are processed according to the relevant taxonomic SOP (standard operating procedure) (available here; Pereira-da-Conceicoa *et al.*, 2022). In summary, the specimen is dissected with different tissues being preserved in separate cryovials according to the relevant taxon SOP. The number of tissue samples generated will depend on specimen size – up to 15 tubes may be required for larger arthropods, for example some larger Odonata. Tissues are also removed for DNA barcoding, prior to the remaining dissection of the specimen – see section 2.5.4. 

Any manipulation of the specimen once removed from the freezer or relevant cold storage unit must be performed directly on dry ice, using a flat surface such as a petri dish to act as a barrier between the specimen and the dry ice. Equipment such as the petri dish or cryotubes should be chilled prior to contact with the specimen.

After destructive sampling (see section 2.6) any equipment used for dissection must be disinfected between samples to minimise cross-sample DNA contamination. A bead heater ( *e.g.*, Fisherbrand Microbead Sterilizer) can be used at 300°C for a duration of 15 seconds for any dissection tools. The dissection tools must have cooled down prior to their use on the frozen specimen. Alternatively, tools may be disinfected with a liquid disinfectant. Gloves must be worn, since human DNA contamination is also possible for steps such as DNA barcoding. Dissected tissue is placed in cryovials (where possible we recommend the use of barcoded cryovials) and stored in freezers at least -80°C before shipment on dry ice to the whole genome sequencing facility.

Each UID will be digitally linked to the barcode number found on the relevant cryovial ( *e.g.* FluidX vials) containing the tissue samples. The body parts and the tissue sample sizes should also be recorded throughout the dissection process, as this information will also be required in the sample manifest.

***Photographing the specimen.*** Before dissection on dry ice, a high-resolution photograph of the specimen should be taken with the corresponding UID, to act as an electronic voucher, as well as to serve as diagnostic support. This photograph (together with any taken while the specimen was alive) serves as a virtual voucher, to potentially verify or support the identification.

***Taking samples for DNA barcoding.*** A small tissue sample is dissected from the specimen and placed either directly into a well in a 96-well PCR plate, in 100% ethanol (100µL per well), or stored in 100% ethanol for future plating (as described in the taxon-specific SOP). For arthropods this tissue sample is typically one, or several, legs, depending on overall specimen size. Whilst barcoding plates do not need to be kept at -80°C, maintenance at low temperatures (typically -25°C) is preferable. See section 2.6 for the barcoding process.

***Taking samples for whole genome.*** The remaining parts of the specimen are then divided into sections (as described in the taxon-specific SOP), with each section in a separate cryovial. Each vial should have a roughly lentil-sized piece of tissue (3–5 mm) in order to produce a whole genome sequence. However, it is worth noting with smaller specimens this is not possible so it will be up to the processor to dissect the specimen appropriately, either by freezing the whole organism or by minimising the number of incisions.

***Physical vouchers.*** If the SOP states that a physical voucher of the specimen must also be preserved (for example, certain segments in Chilopoda and Diplopoda species, or genitalia for various insect groups) this can be stored in a separate vial either in a cool, dry place (if pinning is required) or in 70–90% ethanol. This voucher should also be labelled with the same UID. Ideally, each species should have at least five specimens dissected and processed, to allow for potential failures.

***Specimen preservation.*** The physical specimen must be stored in a facility at -80°C or lower. If the sampled specimen is subsequently transported for whole genome sequencing, depending on the DNA barcoding results, it must be transported at -80°C. This can be by courier licensed to ship specimens on dry ice or in a dry shipper. Under transport regulations dry ice is classified as ‘Dangerous Goods’ and must be labelled and packed accordingly.

### Species and sample data verification

***Barcoding.*** As discussed above, DNA barcoding acts as a tool for identification verification, and has potential to be used for sample tracking at the genome sequencing centre, if a sample switch error is suspected. Once samples for barcoding are received at the barcoding hub, DNA is liberated using a standard overnight lysis protocol, and the lysate is used as a template in the PCR-amplification of the COI marker region ( Hebert *et al.*, 2003). For arthropods, the LCO1490/HCO2198 ( Folmer *et al.*, 1994) and Lep-F1/Lep-R1 ( Hebert *et al.*, 2003) primers are used, with an amplification success rate of typically 90%.

Following PCR product clean-up, and Sanger sequencing, merged forward and reverse sequences are checked for quality, and compared to the BOLD database. In the majority of cases (77.7%), the query sequence has an exact (>99%) match to a species with the same name in the database. A small number of samples fail at either the PCR (8%) or sequencing (14.5%) stage. For the remainder, when provisional DToL identifications do not match suggested taxonomy in BOLD, we need to resolve whether the mismatch is due to (1) DToL misidentification or sample switch error, (2) misidentifications of specimens sequenced and uploaded to BOLD, or (3) because the BOLD specimen has used a different species taxonomy to the DToL specimen. A key approach we use in such cases is to build a phylogenetic tree using the barcode sequence and all similar sequences from the BOLD database (typically the genus). In several cases this approach has resolved or confirmed the identity of a specimen; for example, when the initial sequence identity-based approach was misled by misidentification of a specimen previously uploaded to BOLD. The phylogenetic approach has also highlighted areas of taxonomic uncertainty, such as failure of a CO1 barcode to separate species named from morphology ( Boyes *et al.*, 2021).

***Resolving taxonomy and use of taxon ID numbers for inter-project compatibility.*** Names of organisms are tracked and surfaced through COPO (Collaborative OPen Omics), EBI (European Bioinformatics Institute) and other databases and institutes using the taxonomy in NCBI (National Center for Biotechnology Information). Sometimes the names used in NCBI conflict with those used in the NHM collection management system and any mismatches must be resolved before sample submission. Usually these changes are the result of NCBI using outdated names or combinations but changes are frequently required in the NHM’s CMS taxonomy.

## Concluding remarks

Such a large-scale project naturally poses challenges. We conclude here with a brief discussion of the challenges faced so that future work may glean useful insights.

1. We could not have anticipated that a global pandemic would mean most of us working at home for much of the first year of the project. The value of Wytham Woods as a genomic observatory was apparent then, with sampling nearly uninterrupted and providing the vast majority of DToL samples in the early stages of the project. Sampling by NHM and other partners was severely curtailed, although opportunistic sampling produced specimens (mostly from gardens) which are now resulting in genomes. Besides reduced numbers of specimens, and of a reduced taxonomic diversity, compared to our predictions, the pandemic also resulted in significant disruption to methods developments as we resorted to stop-gap methods. It is important to start a project with a good model for data flow as well as specimen processing and storage.
2. Data connectivity - With such a large project involving multiple collecting organisations, maintaining up-to-date records of species lists and collection progress is challenging. Arthropods in particular represent a huge taxon with many thousands of species and the sequencing process involves several stages. Attempts have been made to keep ‘live’ lists of species as they are collected and progress through the sequencing pipeline. This has, however, been difficult to achieve as there is a trade-off between how useful these lists are and the time it takes to curate them.
3. Developing processes - The huge diversity of taxa within arthropods means that a single collecting, preservation and sequencing process will not be universally suitable. Many groups had not been sequenced before and required specialised processes. As these adaptations to the protocols were novel, and required some degree of trial and error in their development. For example, some species of Lepidoptera are exceedingly short lived and have rapid mortality once collected. Specimens of these species needed to be collected at a genome observatory site and taken directly to laboratory facilities where they could be flash frozen.
4. Sample transport is key when the cold chain cannot be broken. Reliable couriers with expertise in transporting biological samples are necessary and back-up plans are always needed in case dry ice does not arrive in time, or dry shippers malfunction.
5. Where relevant, the heterogametic sex should be prioritised to ensure a more complete representation of the genome. In haplodiploid taxa (mainly Hymenoptera), male genomes are easier to assemble because they are haploid but males are often more difficult to identify than females.
6. Various small-bodied taxa (Collembola, Acari, Nematocera, Diplura, etc.) have proved very challenging to identify live so R&D for these groups has been left for the next phase of DToL, where R&D is also needed to successfully generate long and linked reads from these smaller quantities of DNA.
7. There is an overarching risk of picking ‘low-hanging fruit’ so that many target taxa are ultimately under-represented; discipline is needed to avoid this.
8. Engaging the wider community of naturalists is essential to the long-term success of DToL, therefore these relationships need to be cultivated from a relatively early stage, but expectations also need to be managed. One way in which this has been developed is by including the specimen collector as a named author on the resulting genome note publication, alongside the opportunity for the collector to contribute to writing the genome note.
9. The DNA barcode reference library for British and Irish organisms is still far from complete and requires significant investment. Although any specimens resulting from this project that are subsequently deemed unsuitable for whole genome sequencing can be DNA barcoded (providing we have an accurate ID), we are still a long way away from having a comprehensive DNA barcode reference library, especially for understudied and under recorded smaller-bodied groups such as Collembola or Thysanoptera. Identification by barcodes will become increasingly critical as more of these types of taxa are sampled.
10. Interpreting DNA barcoding results is aided by a phylogenetic approach. Often similarity scores with existing barcodes in the database are insufficient for a conclusive identification. A phylogenetic tree of the barcode gene should ideally be constructed and inspected to infer relationships with the related species concepts.
11. There is a need for ‘virtual voucher’ specimens, where a DNA barcode sequence and high quality photographs act to provide the best possible evidence of the specimen’s identification. To achieve this, new techniques in photography of live and often very small specimens are needed.
12. As the project progresses, the scale of specimen acquisition will need to increase substantially, at the same time as the difficulty in collection and identification is also increasing (as the ‘low-hanging fruit’ will have already been collected). Alternative collection methods may play an important role in addressing this challenge. For example, mass sampling and meta-sequencing.
13. As we venture into sampling different groups, additional, and more detailed, SOPs will be required.

## Data Availability

No data are associated with this article.

## References

[ref-1] Agassiz DJL Beavan SD Heckford RJ : A Checklist of the Lepidoptera of the British Isles. Handbooks for the Identification of British Insects. Field Studies Council on behalf of the Royal Entomological Society, Telford.2013. Reference Source

[ref-2] Boyes D Crowley L Mulhair P : Brave new world: how DNA barcoding is advancing our understanding of insects. *Atropos.* 2021;69:17–30.

[ref-3] Dale-Skey N Askew RR Noyes JS : Checklist of British and Irish Hymenoptera - Chalcidoidea and Mymarommatoidea. *Biodivers Data J.* 2016; (4):e8013. 10.3897/BDJ.4.e8013 27346954 PMC4910507

[ref-5] Folmer O Black M Hoeh W : DNA primers for amplification of mitochondrial cytochrome c oxidase subunit I from diverse metazoan invertebrates. *Mol Mar Biol Biotechnol.* 1994;3(5):294–9. 7881515

[ref-6] Hebert PDN Cywinska A Ball SL : Biological identifications through DNA barcodes. *Proc Biol Sci.* 2003;270(1512):313–21. 10.1098/rspb.2002.2218 12614582 PMC1691236

[ref-7] Invertebrate Link (Joint Committee for the Conservation of British Invertebrates): A code of conduct for collecting insects and other invertebrates. *British Journal of Entomology and Natural History.* 2002;15:1–6. Reference Source

[ref-8] Klopfstein S Kropf C Baur H : *Wolbachia* endosymbionts distort DNA barcoding in the parasitoid wasp genus *Diplazon* (Hymenoptera: Ichneumonidae). *Zool J Linn Soc.* 2016;177(3):541–557. 10.1111/zoj.12380

[ref-9] Lawniczak MKN Davey RP Rajan J : Specimen and sample metadata standards for biodiversity genomics: a proposal from the darwin tree of life project [version 1; peer review: 2 approved with reservations]. *Wellcome Open Res.* 2022;7(187):187. 10.12688/wellcomeopenres.17605.1

[ref-10] Lewin HA Robinson GE Kress WJ : Earth BioGenome Project: Sequencing life for the future of life. *Proc Natl Acad Sci U S A.* 2018;115(17):4325–4333. 10.1073/pnas.1720115115 29686065 PMC5924910

[ref-11] Liston AD Knight GT Sheppard DA : Checklist of British and Irish *Hymenoptera* - Sawflies, *‘Symphyta’*. *Biodivers Data J.* 2014;2:e1168. 10.3897/BDJ.2.e1168 25197241 PMC4152835

[ref-12] Pereira-da-Conceicoa LL Sivell O Sivess L : DToL taxon-specific Standard Operating Procedure for the Terrestrial and Freshwater Arthropods Working Group.protocols.io.2022. 10.17504/protocols.io.261gennyog47/v1

[ref-13] Raper C : United Kingdom Species Inventory (UKSI).Version 37.9. *Natural History Museum.* Checklist dataset,2021. 10.15468/rm6pm4

[ref-14] Sann M Niehuis O Peters RS : Phylogenomic analysis of Apoidea sheds new light on the sister group of bees. *BMC Evol Biol.* 2018;18(1):71. 10.1186/s12862-018-1155-8 29776336 PMC5960199

[ref-15] The Darwin Tree of Life Project Consortium: Sequence locally, think globally: The Darwin Tree of Life Project. *Proc Natl Acad Sci U S A.* 2022;119(4):e2115642118. 10.1073/pnas.2115642118 35042805 PMC8797607

