# Supplementary material for: A sampling strategy for genome sequencing the British terrestrial arthropod fauna
Source: Wellcome Open Res. 2023 Mar 20;8:123. [Version 1] doi: 10.12688/wellcomeopenres.18925.1 (PMC10318377; doi:10.12688/wellcomeopenres.18925.1)
Supplement: Peer review history

# Peer review: Reviewer response for version 1

**DOI:** 10.21956/wellcomeopenres.20983.r60372

**Published:** 2023-07-03

**Authors:** Yasuko Akiyama-Oda

**Affiliations:** 1 Osaka Medical and Pharmaceutical University, Takatsuki, Osaka, Japan

The paper describes a sampling strategy for genome sequencing of arthropods, which is performed in the first phase of a magnificent project called the Darwin Tree of Life project, which aims to determine the genome sequences of all eukaryotic species in Britain and Ireland. The paper details a plan of pre-sequencing processes: compilation of the species list with reasons for inclusion and un-inclusion in the list for sequencing and selection of prioritizing species; two approaches of sample collection and their advantages and disadvantages; specimen identification; and preservation and processing of specimens for DNA barcoding and genome sequencing. In the final section, the authors discuss challenges to resolve in the future studies.

 Overall, the manuscript is written thoroughly and thoughtfully with detailed procedures sufficiently provided.

 A unique aspect of this study is that it engages skills and knowledges of ‘amateur’ researchers especially at the step of sample collection and identification. As evident in Fig. 3, this engagement facilitates sampling of specimens. The aspect is interesting, showing a way of current/future science. The paper mentions what is needed from legal and ethical viewpoints as well as practically for preservation and transport of samples, when people around the country contribute to the project.

 Comments:

- In the Introduction section, the authors write that each genome is derived from one specimen whenever possible. I agree the importance of obtaining data from one specimen; this could make an assemble step easier. However, there could be differences in the individual level (e.g., SNPs, mutations, chromosome-level differences), and potentially other factors (e.g., contaminants as food, infection) could also affect data interpretation. It is difficult to determine representative one from natural sources. The idea and treatment of individual differences should be discussed.

The minor point:

- The abbreviation SOP appears before it is fully written.

Does the article adequately reference differing views and opinions?

Yes

Are all factual statements correct, and are statements and arguments made adequately supported by citations?

Yes

Is the Open Letter written in accessible language?

Yes

Where applicable, are recommendations and next steps explained clearly for others to follow?

Yes

Is the rationale for the Open Letter provided in sufficient detail?

Yes

Reviewer Expertise:

Developmental Biology of arthropods

I confirm that I have read this submission and believe that I have an appropriate level of expertise to confirm that it is of an acceptable scientific standard.

# Peer review: Reviewer response for version 1

**DOI:** 10.21956/wellcomeopenres.20983.r55660

**Published:** 2023-03-30

**Authors:** Marko Prous

**Affiliations:** 1 Department of Zoology, Institute of Ecology and Earth Sciences, Tartu Ulikool, Tartu, Tartu County, Estonia

The article describes terrestrial arthropod sampling strategy, specimen identification (including COI barcoding) and processing for the ambitious Darwin Tree of Life (DToL) project. Mostly it describes the reasons for the selection of species and sampling sites in the initial phase of the project, often determined by various practicalities (minimum specimen size, scientific interests, collecting permits, proximity of sampling sites to laboratories). There could be also useful tips for other similar projects in general, for example regarding cold chain (important for DNA preservation for long read sequencing), specimen processing, and barcoding (important also for tracking samples going to genome sequencing centre). The authors also highlight engaging the wider community of naturalists that is essential to any project of that nature.

 There are some typos.

1. Replace "carefµL" (microliter) with "careful" on page 7.
2. Replace "usefµL" with "useful" on page 9. 
3. Replace "sssto" with "to" on page 11.

Some comments:

 The authors write "We have been retaining wings of macrolepidoptera and genitalia of several insect groups, particularly various moths, some Diptera (such as Mycetophilidae) and certain genera of Hymenoptera (e.g., *Netelia*)." - Saws (ovipositors) and genitalia of many sawflies (Hymenoptera) are also important for species identification and should be preserved when possible.

 "DNA barcoding using standard markers does not reliably differentiate species of all genera (e.g., certain ichneumonid wasp genera: Klopfstein *et al.*, 2016)." - This applies also to many sawflies, particularly in the subfamily Nematinae ^1^^,^^2^.

 "Unique identifiers and sample data. Before processing, each specimen is assigned a unique identifier, e.g. a unique specimen ID (UID)." - It should be clarified (for instance giving an example) what this unique specimen ID is. For example, this BioSample in GenBank.

 Which of the many ID numbers given for this BioSample is the unique specimen ID? 6d0b0c23-6fac-462d-9964-e35e452bcd9c? Sample name 614dcf84edc59f2e4a450697? specimen id Ox001515? tolid iyRhoChlo1?

Does the article adequately reference differing views and opinions?

Yes

Are all factual statements correct, and are statements and arguments made adequately supported by citations?

Yes

Is the Open Letter written in accessible language?

Yes

Where applicable, are recommendations and next steps explained clearly for others to follow?

Yes

Is the rationale for the Open Letter provided in sufficient detail?

Yes

Reviewer Expertise:

Taxonomy and phylogeny of sawflies (Hymenoptera).

I confirm that I have read this submission and believe that I have an appropriate level of expertise to confirm that it is of an acceptable scientific standard.

## References

[1] : North-Western Palaearctic species of Pristiphora (Hymenoptera, Tenthredinidae). *Journal of Hymenoptera Research* .2017;59: 10.3897/jhr.59.12565 1-190 10.3897/jhr.59.12565

[2] : Cross-contamination and strong mitonuclear discordance in Empria sawflies (Hymenoptera, Tenthredinidae) in the light of phylogenomic data. *Mol Phylogenet Evol* .2020;143: 10.1016/j.ympev.2019.106670 106670 10.1016/j.ympev.2019.106670 31706020
